# Supplementary material for: Parkin overexpression attenuates muscle atrophy and improves mitochondrial bioenergetics but not histological features of Duchenne muscular dystrophy in mice
Source: Sci Rep. 2026 Jan 17;16:4139. doi: 10.1038/s41598-025-34223-9 (PMC12858866; doi:10.1038/s41598-025-34223-9)

Figure S1: Representative recordings to assess (A) mitochondrial respiration rates and (B) H_2O_2 emission rates. In (B), only stable portions of fluxes were selected, and artifacts induced by additions of chemicals were excluded.

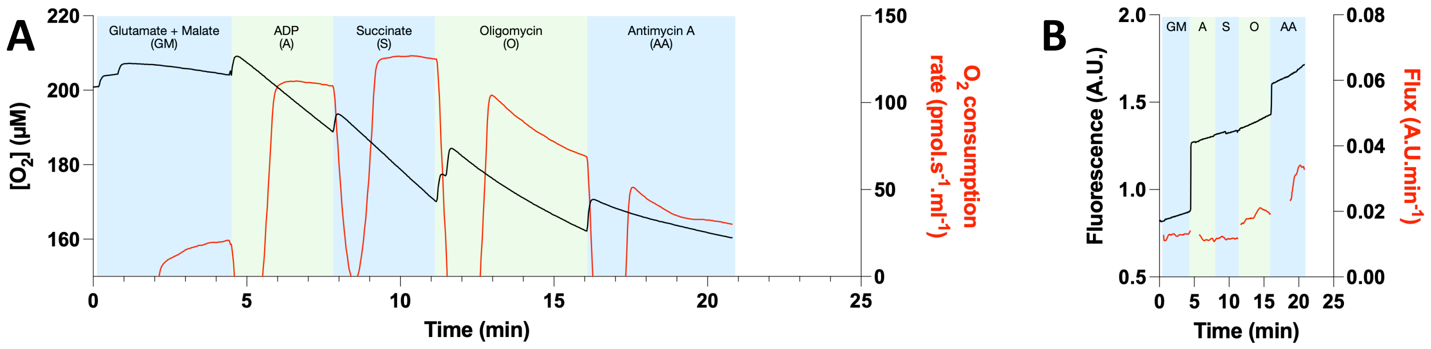

Figure S2: Original immunblots and stain free images presented in Figures 1 and 2

Figure 1B: Immunoblot for Parkin (left) and corresponding stain free image (right; loading control)

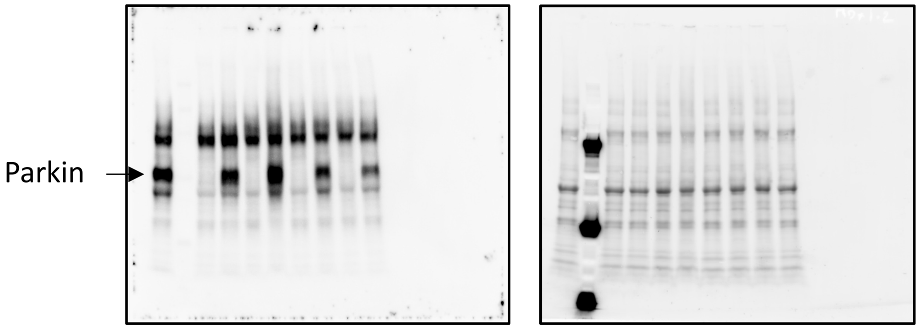

Figure 2E: Immunoblot for representative OXPHOS subunits (left) and corresponding stain free image (right; loading control)

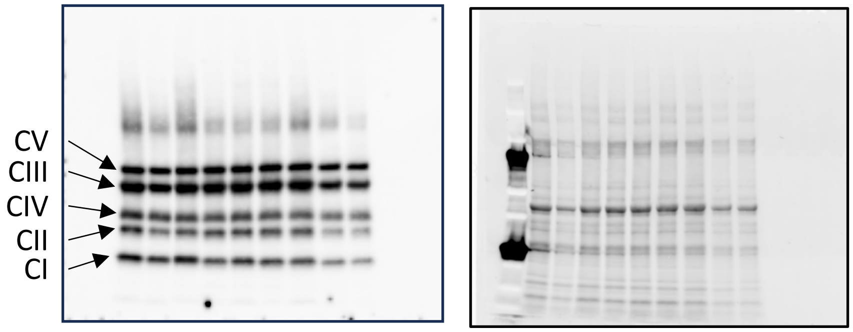

Figure 2F: Immunoblot for representative TFAM (first row), ANT (second row), VDAC1 (third row) and corresponding stain free image (right; loading control)

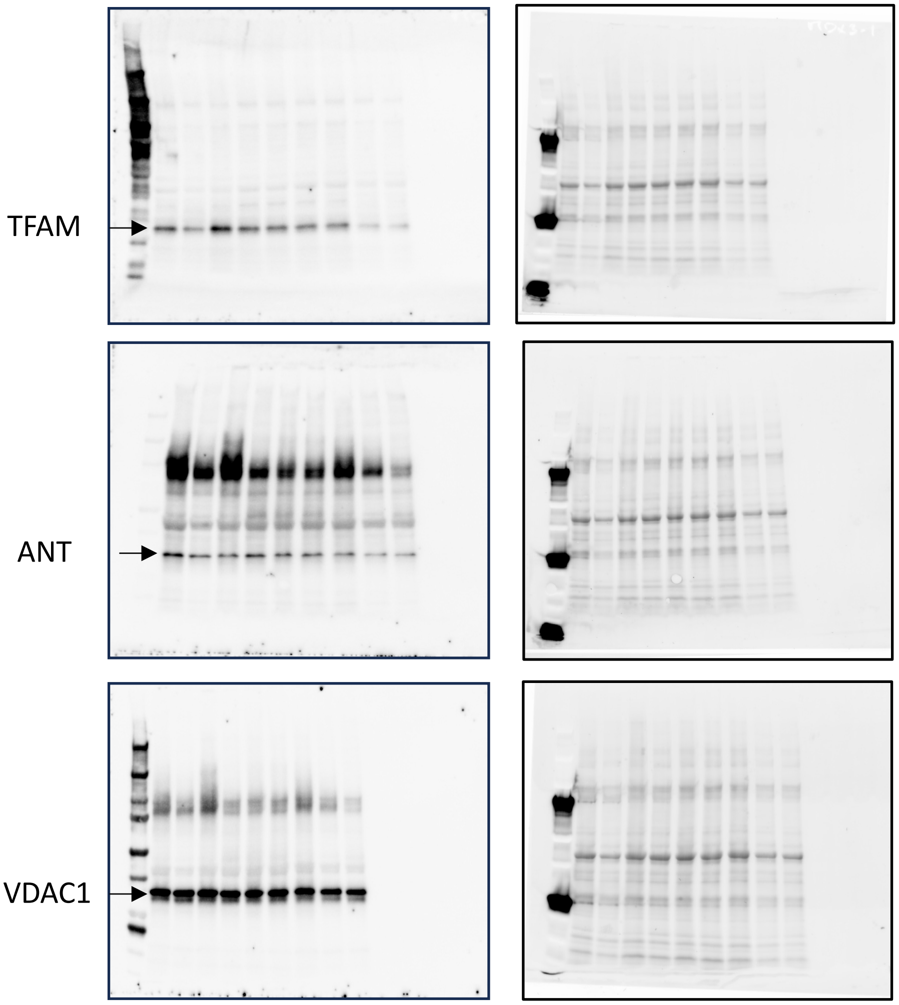

Figure S3: Original immunblots and stain free images presented in Figures 3 and 5

Figure 3B: Immunoblot for Parkin (left) and corresponding stain free image (right; loading control)

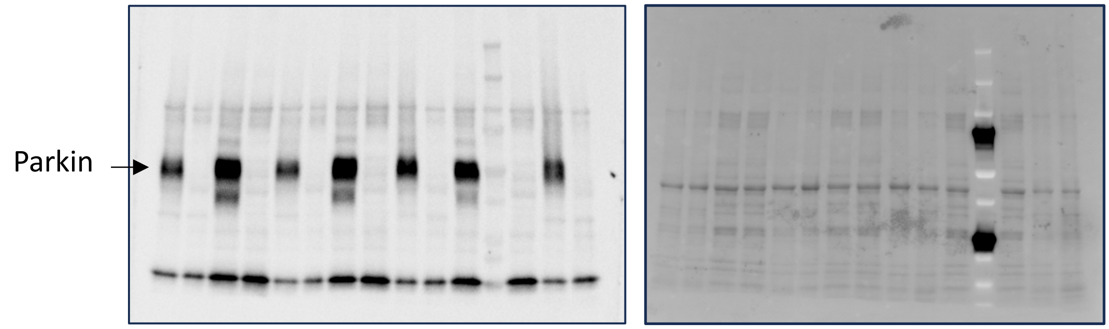

Figure 5A: Immunoblot for TOM20 subunits (left) and corresponding stain free image (right; loading control)

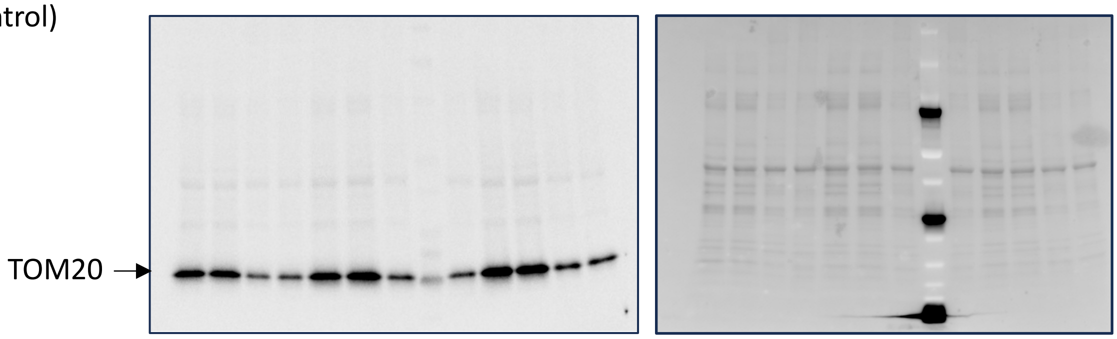

Figure 5B: Immunoblot for VDAC1 subunits (left) and corresponding stain free image (right; loading control)

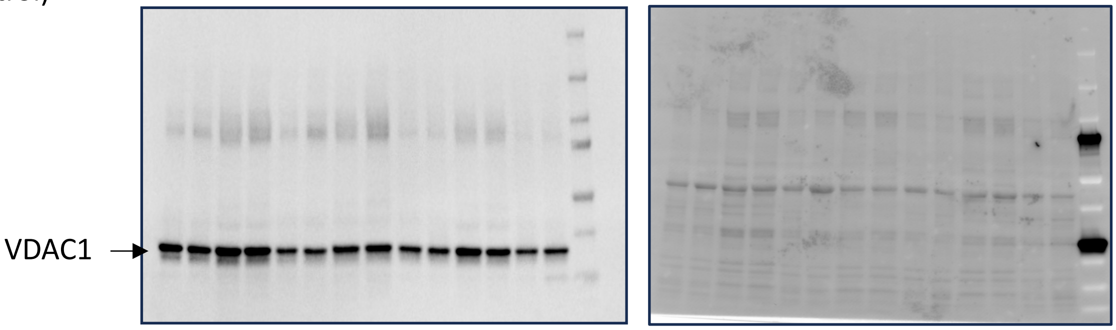

Supplement: Supplementary file 1 — Supplementary Material 1 [file 41598_2025_34223_MOESM1_ESM.pdf]
